# One-step fermentation for producing xylo-oligosaccharides from wheat bran by recombinant *Escherichia coli* containing an alkaline xylanase

**DOI:** 10.1186/s12896-022-00736-8

**Published:** 2022-02-05

**Authors:** Jiawen Liu, Cong Liu, Shilei Qiao, Zhen Dong, Di Sun, Jingrong Zhu, Weijie Liu

**Affiliations:** Jiangsu Key Laboratory of Phylogenomics and Comparative Genomics, School of Life Science, Jiangsu Normal University, No. 101, Shanghai Road, Tongshan District, Xuzhou, 221116 Jiangsu Province China

**Keywords:** Prebiotics, *Bacillus agaradhaerens*, Single-step fermentation, Xylanase, Response surface optimization

## Abstract

**Background:**

One-step fermentation is a cheap way to produce xylo-oligosaccharides (XOS), where production of xylanases and XOS is integrated into a single process. In spite of cost advantage, one-step fermentation is still short in yield so far due to the limited exploration. To cope with this issue, production of XOS from wheat bran by recombinant *Escherichia coli* through one-step fermentation was investigated in this study.

**Results:**

An endo-β-1,4-xylanase gene belonging to glycoside hydrolase family 11 of *Bacillus agaradhaerens* was employed to construct recombinant *E. coli*. This xylanase showed maximal activity at 60 °C and pH 8.0–8.5. Its activity retained more than 60% after incubation at 70 °C for 4 h, showing a good stability. The recombinant *E. coli* BL21(DE3) could secreted xylanases that directly hydrolyzed de-starched wheat bran to XOS in fermentation medium. The XOS generated from hydrolysis consisted of xylose, xylobiose and xylotriose accounting for 23.1%, 37.3% and 39.6%, respectively. Wheat bran concentration was found to be the most crucial factor affecting XOS production. The XOS concentration reached 5.3 mg/mL at 10% loading of wheat bran, which is higher than those of previous researches. Nitrogen source type could also affect production of XOS by changing extracellular xylanase activity, and glycine was found to be the best one for fermentation. Optimal fermentation conditions were finally studied using response surface optimization. The maximal concentration emerged at 44.3 °C, pH 7.98, which is affected by characteristics of the xylanase as well as growth conditions of *E. coli*.

**Conclusions:**

This work indicates that the integrated fermentation using recombinant *E. coli* is highly competitive in cost and final concentration for producing XOS. Results can also provide theoretical basis for large-scale production and contribute to the wide adoption of XOS.

**Supplementary Information:**

The online version contains supplementary material available at 10.1186/s12896-022-00736-8.

## Background

Prebiotics, namely some kinds of oligosaccharides, can specifically promote the activity of beneficial bacteria in gastrointestinal tract [1, 2]. With insight into the effect of gut microbiota on human overall health, prebiotics have been the hotspot in consumption and research currently [3]. Xylo-oligosaccharides (XOS) are emerging probiotics which consist of several β-1,4 linked xylose units [4]. They have been attached importance to recent years due to remarkable prospect of application in food, medicine, poultry and other fields [5–7]. Furthermore, XOS are more efficient than other prebiotics in enhancing growth of certain bifidobacteria and in protecting lactobacilli under stress environments [8–10]. XOS also present good heat and pH stability, which is beneficial to retaining more nutritional properties in digestive tract [11]. Because of these advantages, market demand for XOS is rising quickly and expected to reach 130 million U.S. dollars in 2023 at an annual growth of 5.3% [12].

Enzymatic hydrolysis is one of the major methods to produce XOS, which is more environmentally friendly and generates less undesired by-products than chemical hydrolysis [13]. Xylanases are the critical factor for enzymatic production of XOS, which act on backbone of xylan and convert it into XOS as well as xylose. Xylanases belonging to glycoside hydrolase (GH) family 11 attack unsubstituted sites of xylan, whose hydrolysate mainly consists of xylobiose and xylotriose; GH10 xylanases can accommodate a decorated xylopyranosyl residue at − 1 subsite, resulting in production of both linear and substituted XOS with low degree of polymerization (DP); GH30 xylanases prefer branched xylan than the linear one so substituted XOS are their principal products [14, 15]. Hydrolyzing extracted xylan or raw lignocellulosic biomass using these xylanases to produce XOS has been widely reported, and XOS yields are very attractive in some works [16, 17]. However, preparation of these purified enzymes is unwieldy and costly. In addition, high temperature is commonly needed for an efficient enzymatic hydrolysis, which also prejudices the cost of production process [18, 19]. To cope with these issues, some researches devoted to integrating production of xylanases and XOS into a single process. In such process, microorganisms extracellularly secrete xylanases and meanwhile, these enzymes directly convert xylan or lignocellulosic biomass into XOS in medium. For example, a wild-type *Bacillus subtilis* was reported to produce XOS by direct fermentation utilizing brewers’ spent grain, and XOS could yield further increase when *B. subtilis* was genetically modified [20]. Some fungi, such as *Trichoderma reesei* and *Aspergillus nidulans*, exhibited potential of producing XOS in one-step fermentation as well [21, 22]. These integrated production of XOS left out separate process for preparing xylanases, and generally adopted mild fermentation conditions, which contributes to overcoming cost challenge [12]. Nevertheless, XOS yields of one-step fermentation are commonly disadvantaged comparing with those of enzymatic hydrolysis. Indeed, yields can be improved by optimizing types of medium, substrates, fermentation microorganisms and conditions, but researches about these issues are scarce. For example, only a few bacillus and fungi are employed for one-step fermentation so far. These microorganisms, however, can all utilize XOS as carbon source, which prejudices the accumulation of XOS in medium. In addition, critical restriction limiting XOS production remains unknown for such integrated fermentation, which leads to difficulty in substantial improvement in yield.

*Escherichia coli* has been used to produce food additives and drugs for decades, which has been proved to be safe and reliable [23, 24]. *E. coli* is probably suited to fermentation for producing XOS because it cannot consume this kind of oligosaccharides [25]. However, feasibility of one-step fermentation employing *E. coli* lacks sufficient study. Wheat bran is a xylan-rich by-product of white flour milling and has been used as cheap raw materials for XOS production previously [26, 27]. *Bacillus agaradhaerens* C9 is an alkaliphilic strain with lignocellulose-degrading ability. Secretion of alkali-tolerant xylanases by *B. agaradhaerens* C9 was verified in our previous work [28]. Bioinformatics analysis of its genome revealed an GH11 xylanase that was named *Ba*xyl11. In this study, *Ba*xyl11 was expressed using *E. coli* BL21(DE3) and enzymatic characteristics of recombinant *Ba*xyl11 (r*Ba*xyl11) were then investigated. Moreover, producing XOS from wheat bran by the recombinant *E. coli* BL21(DE3) containing r*Ba*xyl11 was carried out. Effects of wheat bran concentration, nitrogen source type and fermentation conditions (pH and temperature) on XOS production were finally investigated. These results would contribute to overcoming yield and cost challenge in the production of XOS, and promote its wide adoption.

## Results

### Enzymatic characteristics of r*Ba*xyl11

**Fig. 1:**
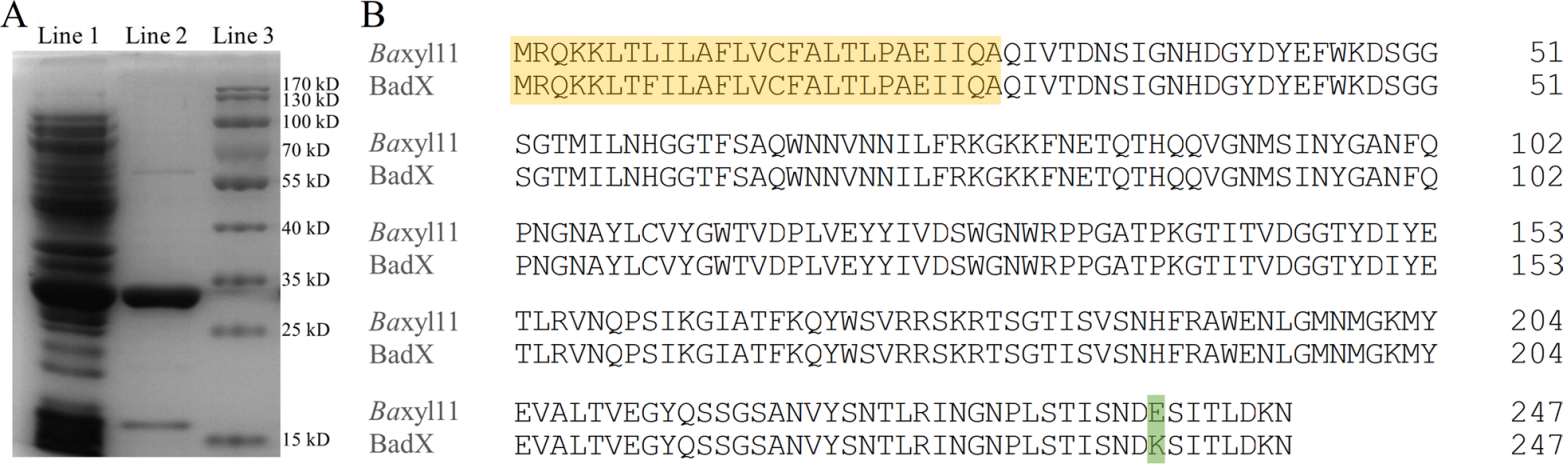
Electrophoresis and sequence analysis of r*Ba*xyl11. **a** Sodium dodecyl sulfate polyacrylamide gel electrophoresis analysis of r*Ba*xyl11. Line 1: soluble cell extract containing r*Ba*xyl11; Line 2: r*Ba*xyl11 after purification; Line 3: marker. **b** Sequence alignment of *Ba*xyl11 and BadX. Amino acid residues belonging to signal peptide are marked with yellow background. Different amino acid residues between *Ba*xyl11 and BadX are marked with green background

*Ba*xyl11 gene was cloned from genomic DNA of *B. agaradhaerens* C9 and ligated with plasmid pET22b(+). r*Ba*xyl11 was then expressed using *E. coli* BL21(DE3). The purified r*Ba*xyl11 showed electrophoretic homogeneity and its molecular weight corresponded to the calculated value of 28.9 kD (Fig. 1a). r*Ba*xyl11 presented hydrolytic activity to glucuronoxylan and arabinoxylan but not to cellulose, mannan, starch and 4-nitrophenyl-beta-D-xylopyranoside (pNPX), which demonstrated that r*Ba*xyl11 is an endoxylanase.

To evaluate its catalytic activities, kinetic parameters of r*Ba*xyl11 against arabinoxylan and glucuronoxylan were measured (Table 1). V_max_ and K_cat_ against arabinoxylan were approximately two times as high as those against glucuronoxylan, showing higher activity against arabinoxylan. However, lower K_m_ against glucuronoxylan indicated the preference for such polysaccharide than arabinoxylan. As a result, the K_cat_/K_m_ of r*Ba*xyl11 against glucuronoxylan was higher than that against arabinoxylan.

**Table 1:** Kinetic parameters of r*Ba*xyl11 for xylans

Substrate	V_max_ (μΜ/s)	K_cat_ (/s)	K_m_ (g/L)	K_cat_/K_m_ (L/g/s)
Arabinoxylan	44.2 ± 3.7	599.0 ± 49.7	10.9 ± 0.9	55.0 ± 0.3
Glucuronoxylan	24. 3 ± 0.6	330.1 ± 7.7	4.1 ± 0.1	79.7 ± 1.2

Concentration of r*Ba*xyl11 was 220 nΜ for measurement. All data are presented as mean ± standard deviation (n = 3)

**Fig. 2:**
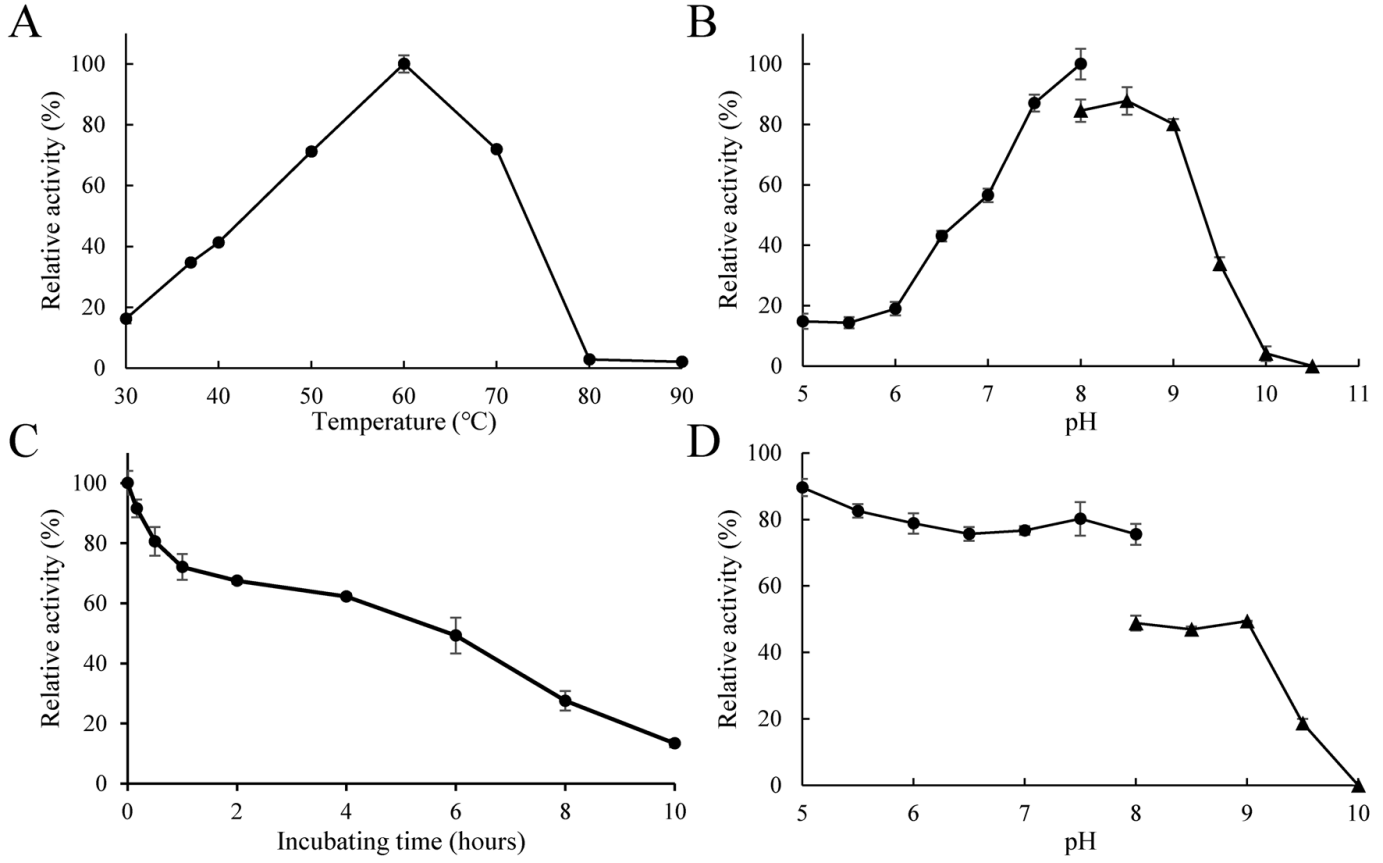
Effect of temperature and pH on activity and stability of r*Ba*xyl11. **a** Effect of temperature on activity of r*Ba*xyl11. **b** Effect of pH on activity of r*Ba*xyl11. **c** Effect of temperature on stability of r*Ba*xyl11. **d** Effect of pH on stability of r*Ba*xyl11. In (**a**) and (**b**), the maximal activity was designated as 100%. In (**c**) and (**d**), activity of enzyme without incubation was designated as 100%. Measurement at pH 5.0–8.0 and 8.0–10.5 was carried out in Na_2_HPO_4_-NaH_2_PO_4_ buffer and Na_2_CO_3_-NaHCO_3_ buffer, respectively. All data are presented as means ± standard deviations (n = 3)

To investigate the optimal conditions for catalysis, activities of r*Ba*xyl11 were measured at different temperatures and pH values (Fig. 2a, b). r*Ba*xyl11 showed highest activity at 60 °C and its optimal pH ranged from 8.0 to 8.5, indicating it is an alkaline xylanase. Stability of r*Ba*xyl11 was then studied (Fig. 2c, d). Activity of r*Ba*xyl11 retained more than 80% after incubation at 70 °C for 30 min, and even after 4 h, 60% of its activity could be maintained. Moreover, r*Ba*xyl11 showed good stability when incubated at the pH ranging from 5.0 to 9.0, which is commonly the appropriate pH range for fermentation.

### One-step fermentation for XOS production

**Fig. 3:**
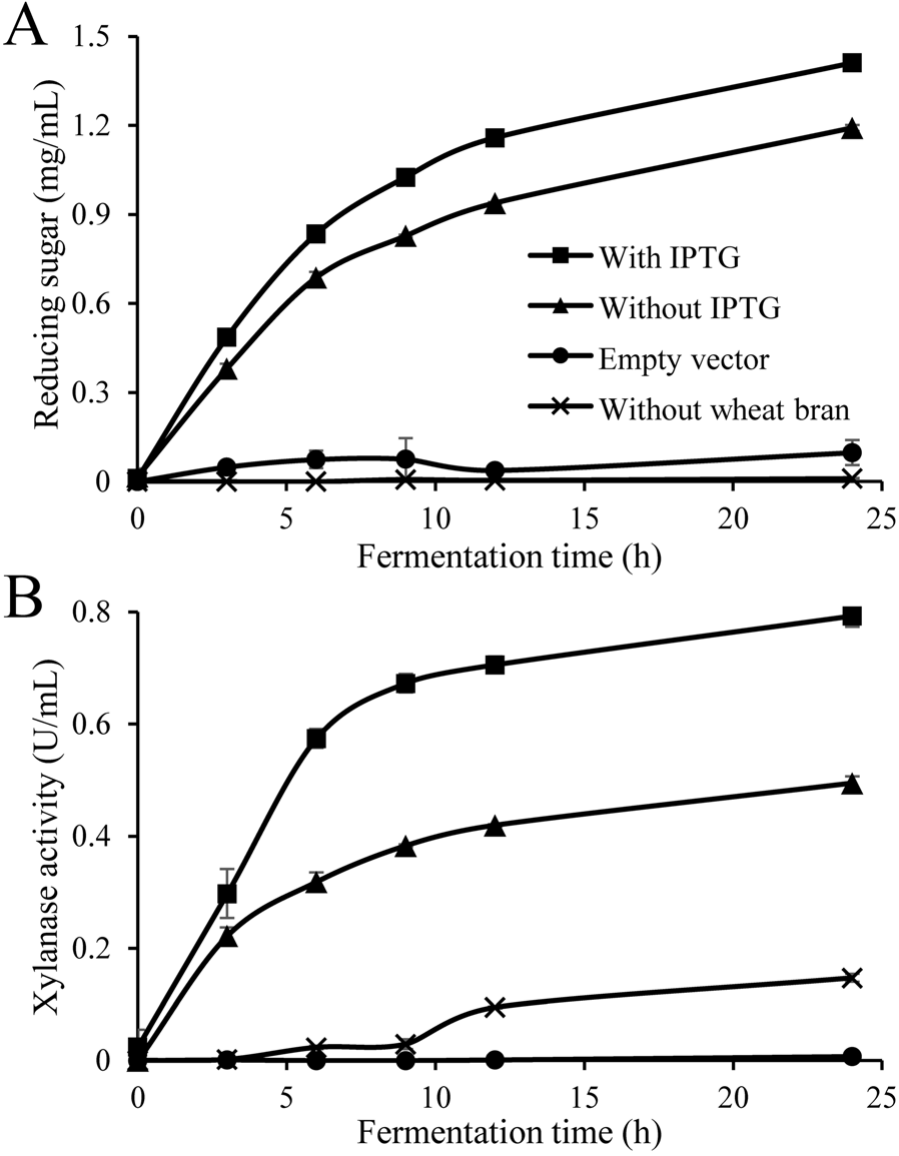
The time course of **a** XOS concentration and **b** extracellular xylanase activity during one-step fermentation. “With IPTG”: employ of inducer (IPTG), wheat bran and recombinant *E. coli* containing r*Ba*xyl11; “Without IPTG”: employ of wheat bran and recombinant *E. coli* containing r*Ba*xyl11 without inducer; “Empty vector”: employ of inducer, wheat bran and recombinant *E. coli* containing unmodified pET22b(+); “Without wheat bran”: employ of inducer and recombinant *E. coli* containing r*Ba*xyl11 without wheat bran. Wheat bran concentration: 2%. All data are presented as means ± standard deviations (n = 3)

To save cost and simplify process, direct fermentation by r*Ba*xyl11-transformed *E. coli* BL21(DE3) to produce XOS from wheat bran was carried out. Starch in wheat bran was removed in advance for a better XOS yield. Employ of the recombinant *E. coli* BL21(DE3) in the presence of isopropyl-1-thio-β-D-galactopyranoside (IPTG) and wheat bran for fermentation resulted in a reducing sugar yield of 1.41 mg/mL at the 24th hour (Fig. 3a). By contrast, fermentation without wheat bran or using *E. coli* BL21(DE3) containing raw plasmid only produced negligible reducing sugars, demonstrating that r*Ba*xyl11 from *E. coli* could produce XOS by acting on wheat bran in such one-step fermentation.

In the presence of r*Ba*xyl11 and wheat bran, xylanase activity increased rapidly in the first 6 h and slowly then (Fig. 3b). Activity in the medium without IPTG showed similar trend but at lower level. It is noteworthy that use of IPTG raised xylanase activity by 40% while only increased XOS concentration by 18%, suggesting that xylanase activity is not the most important factor to yield (see “Effect of nitrogen source type on one-step fermentation” section for details).

### Product composition of r*Ba*xyl11 acting on wheat bran

**Fig. 4:**
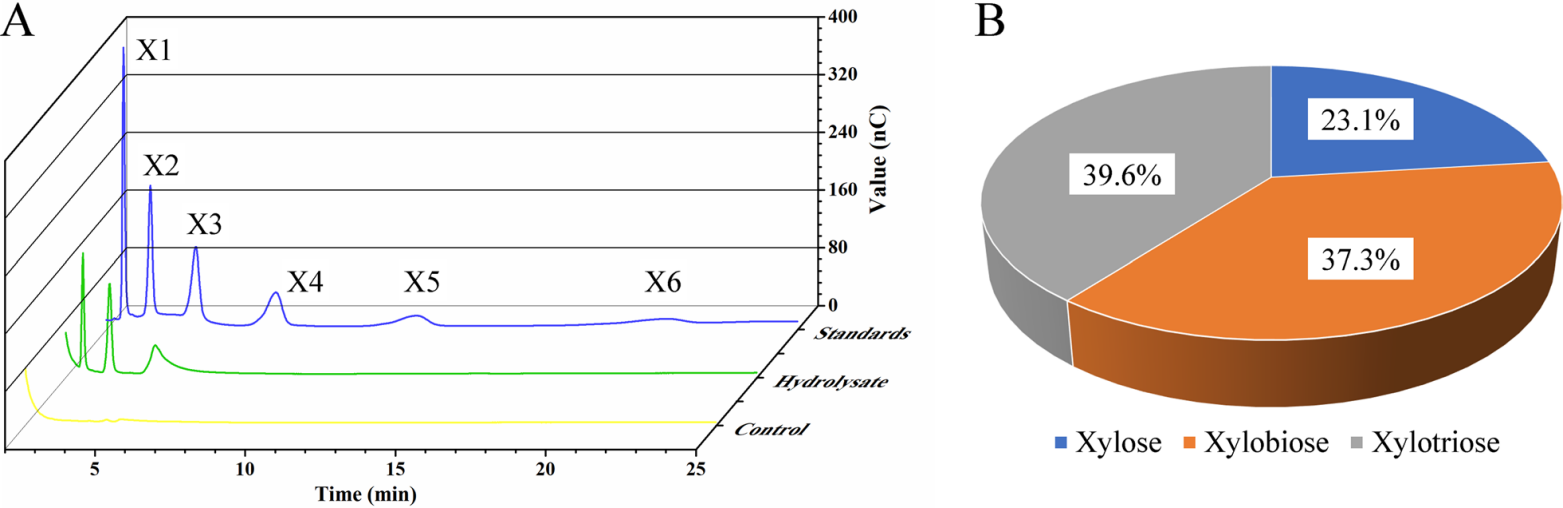
Product composition of r*Ba*xyl11 acting on wheat bran analyzed by HPIC. **a** Product composition of the hydrolysis. “Standards”: mixture of xylose, xylobiose, xylotriose, xylotetraose, xylopentaose and xylohexaose with respective concentration of 2 mg/mL; “Hydrolysate”: XOS produced by r*Ba*xyl11 from wheat bran; “Control”: sample of hydrolysate without employ of r*Ba*xyl11. **b** Quantitative analysis of xylose and XOS produced by r*Ba*xyl11

To study the product composition of r*Ba*xyl11 acting on wheat bran, its hydrolysate was analyzed using high pressure ion chromatography (HPIC). Results demonstrated that xylose, xylobiose and xylotriose are the primary product (Fig. 4a). Further quantitative analysis basing on chromatogram showed that xylose, xylobiose and xylotriose respectively accounted for 23.1%, 37.3% and 39.6% (Fig. 4b). In other words, about 77% of its product is low-DP XOS when r*Ba*xyl11 hydrolyzed wheat bran.

### Effect of wheat bran concentration on one-step fermentation

**Fig. 5:**
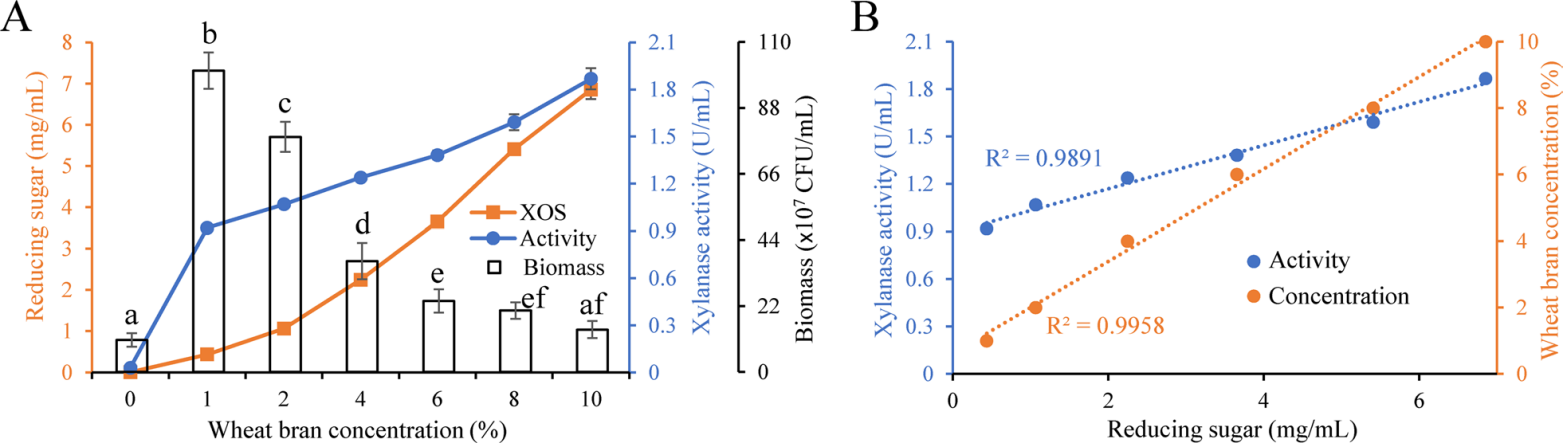
Effect of wheat bran concentration on one-step fermentation. **a** Effect of wheat bran concentration on XOS production, xylanase activity and biomass of recombinant *E. coli*. **b** Correlation between XOS concentration and xylanase activity as well as wheat bran concentration. Fermentation time: 12 h. Temperature: 37 °C. Initial pH: 7.0. In (**a**), data are presented as means ± standard deviations (n = 3)

Effect of wheat bran concentration on XOS yield was investigated here. As showed in Fig. 5a, XOS concentration increased with wheat bran concentration in 0–10% of loading range, and further raise in substrate loading would lead to an excessive viscosity of medium. At 10% of wheat bran concentration, 6.9 mg/mL of reducing sugar was obtained. That means the XOS concentration reached 5.3 mg/mL (excluding xylose), which is very considerable.

To study how wheat bran concentration affects production of XOS, xylanase activity and growth of *E. coli* BL21(DE3) were also measured. Xylanase activity increased with wheat bran concentration while the biomass of *E. coli* BL21(DE3) showed opposite trend, indicating that high-concentration wheat bran stimulated the synthesis and secretion of r*Ba*xyl11 and inhibited the growth of *E. coli* BL21(DE3) (Fig. 5). A high xylanase activity would contribute to XOS production, but the huge increase of final concentration was not exclusively due to this reason. Specifically, XOS concentration increased nearly 16-fold when wheat bran concentration raised tenfold from 1 to 10%, and meanwhile, xylanase activity only increased by 103%. It is obvious that augmentation of XOS yield resulted from combined effect of the increase in both wheat bran concentration and xylanase activity, where the former contributed more. In other words, substrate concentration is the decisive factor to XOS production in such fermentation process instead of enzymatic activity.

### Effect of nitrogen source type on one-step fermentation

**Fig. 6:**
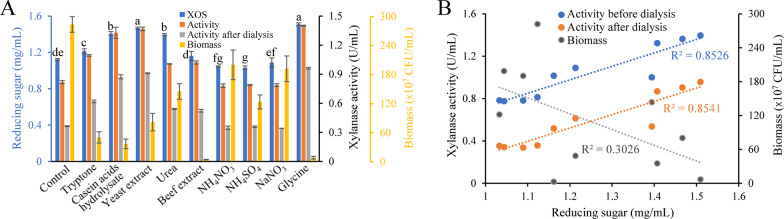
Effect of nitrogen source type on one-step fermentation. **a** Effect of nitrogen source type on XOS production, xylanase activity and biomass of recombinant *E. coli*. “Activity”: xylanase activity measured using supernatant of medium; “Activity after dialysis”: xylanase activity measured using dialysis-treated supernatant of medium. **b** Correlation between XOS concentration and xylanase activity as well as wheat bran concentration. Wheat bran concentration: 2%. Fermentation time: 12 h. Temperature: 37 °C. Initial pH: 7.0. In (**a**), data are presented as means ± standard deviations (n = 3)

Effect of nitrogen source type on one-step fermentation was investigated here (Fig. 6a). Measurement of reducing sugar indicated that maximal XOS concentration was obtained when using glycine as nitrogen source, which is slightly higher than that using yeast extract (p value = 0.065). The lowest three yields showed when NH_4_NO_3_, NaNO_3_ and NH_4_SO_4_ were employed, indicating such inorganic salts are not suited to production of XOS.

The correlation analysis was then conducted to evaluate the effect of nitrogen sources on XOS. To avoid the effect of difference in nitrogen sources on enzymatic activity assay, medium after dialysis was also employed for measurement (Fig. 6b). Results demonstrated a positive linear correlation between XOS concentration and xylanase activity regardless of whether medium was treated with dialysis. By comparison, no credible correlation between XOS concentration and biomass was observed. Therefore, types of nitrogen source affected XOS production mainly by changing xylanase activity.

### Optimizing XOS production by response surface methodology

**Fig. 7:**
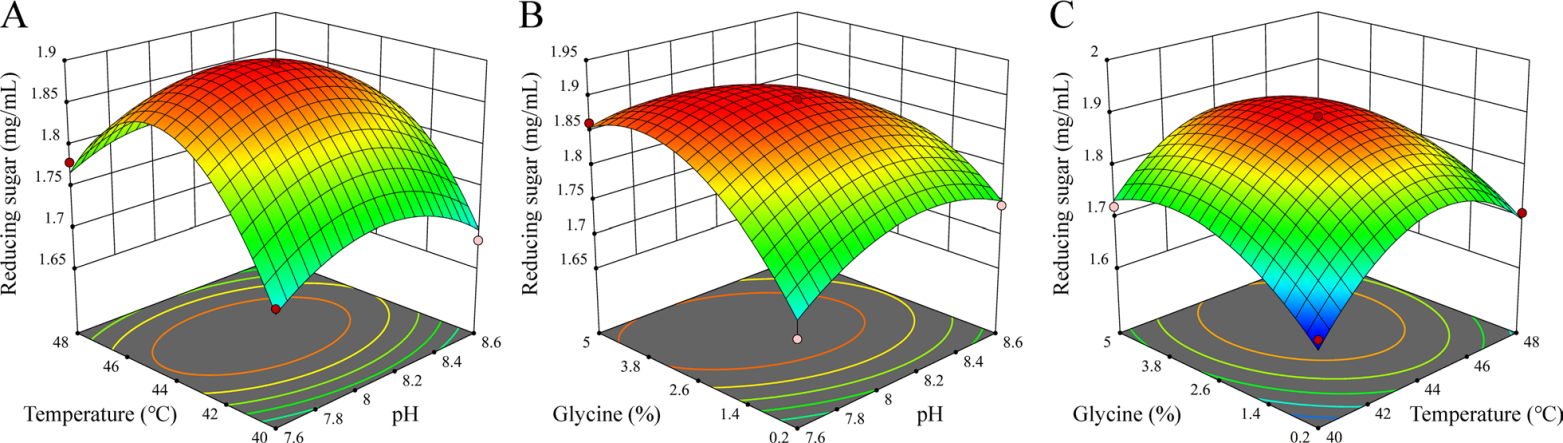
Response surface showing effect of temperature, pH and glycine concentration on XOS production. **a** Effect of temperature and pH on XOS production. **b** Effect of glycine concentration and pH on XOS production. **c** Effect of glycine concentration and temperature on XOS production. Wheat bran concentration: 2%. Fermentation time: 12 h. All experiments were conducted in triplicate

To study effect of fermentation conditions, temperature, pH and glycine concentration were chosen as variables for optimization using Box-Behnken design. After 12-h fermentation, concentrations of reducing sugars varied in the range of 1.629–1.895 mg/mL (Table 2 and Fig. 7). Glycine concentration is the most influential variable with p-value = 0.0006, followed by temperature with p value = 0.0132. It was predicted that the optimal concentration of 1.904 mg/mL would be obtained at 44.3 °C, pH 7.98 with 3.36% of glycine, which corresponds approximately to the central-point condition of the design. The optimal pH for fermentation corresponded to that of catalysis by r*Ba*xyl11 (Fig. 2b), while the optimal temperature is lower than that for catalysis (60 °C) and higher than the best growth temperature of *E. coli* (37 °C).

**Table 2:** Experimental design to study the effect of pH, fermentation temperature and glycine concentration on XOS production

Run	pH	Temperature (°C)	Glycine (%)	Concentration (mg/mL)
1	8.1	40	0.2	1.629 ± 0.008
2	8.1	44	2.6	1.895 ± 0.009
3	7.6	48	2.6	1.780 ± 0.009
4	7.6	44	0.2	1.673 ± 0.014
5	8.1	48	5.0	1.721 ± 0.014
6	8.6	44	5.0	1.784 ± 0.039
7	8.1	40	5.0	1.721 ± 0.013
8	8.1	44	2.6	1.895 ± 0.009
9	8.1	48	0.2	1.709 ± 0.018
10	8.1	44	2.6	1.895 ± 0.009
11	7.6	44	5.0	1.862 ± 0.044
12	8.6	44	0.2	1.743 ± 0.003
13	7.6	40	2.6	1.703 ± 0.011
14	8.1	44	2.6	1.895 ± 0.009
15	8.6	48	2.6	1.714 ± 0.008
16	8.1	44	2.6	1.895 ± 0.009
17	8.6	40	2.6	1.685 ± 0.055

All data are presented as mean ± standard deviation (n = 3)

## Discussion

High cost is a challenge limiting the enzymatic production of XOS. One-step fermentation is a cost-efficient way to produce XOS, but its yield was commonly modest comparing with that of enzymatic hydrolysis (Table 3). For example, hydrolyzing mahogany employing a xylanase of *Clostridium* resulted in a XOS concentration of 4.5 mg/mL [29]. The concentrations could even exceed 8 mg/mL when using extracted xylan as substrate [16]. By comparison, only 0.8–1.1 mg/mL of XOS were obtained employing *B. subtilis* or *Trichoderma* species in one-step fermentation despite optimization [20, 21]. A higher concentration of 3.2 mg/mL was obtained when using wheat middlings and *B. subtilis*, but the fermentation time, 48 h, was less competitive [30]. This study described a considerable XOS concentration of 5.3 mg/mL with only 12-h fermentation, which is much higher than those of previous works. Moreover, substrate concentration was found to be the most influential factor to XOS production here. This is probably the cause of modest XOS yields in previous reports because XOS yield were prejudiced by a large loading of substrates using *B. subtilis* and fungi [20, 21]. Therefore, employing *E. coli* BL21(DE3) is promising to eliminate the disadvantage in XOS production by one-step fermentation.

**Table 3:** XOS production by enzymatic hydrolysis and one-step fermentation

Substrate	Enzyme or strain	Reaction time (h)^a^	XOS yields^b^		XOS production method	References
			mg/mL^c^	mg/g substrate^d^		
Wheat bran	Engineering *E. coli* BL21(DE3)	12	0.8–5.3^e^	53-80^e^	One-step fermentation (37 °C)	This study
Wheat middlings	*Bacillus subtilis*	48	3.2^e^	64^e^	One-step fermentation (37 °C)	[30]
Brewers’ spent grain	Engineering *Bacillus subtilis*	12	1.1	34	One-step fermentation (45 °C)	[20]
Brewers’ spent grain	*Trichoderma reesei*	72	0.8	40	One-step fermentation (30 °C)	[21]
Rice husk	Engineering *Aspergillus nidulans*	48	–	24	One-step fermentation (37 °C)	[22]
Pistachio shell	Commercial xylanase	10	2.7^e^	–	Enzymatic hydrolysis (45 °C)	[43]
Mahogany	Xylanase from *Clostridium* strain BOH3	24	4.5^e^	90^e^	Enzymatic hydrolysis (50 °C)	[29]
Sugarcane bagasse	Xylanase from *Bacillus subtilis*	15	3.6	119	Enzymatic hydrolysis (50 °C)	[44]
Rice straw	Commercial xylanase	24	0.1^e^	2^e^	Enzymatic hydrolysis (50 °C)	[45]
Rice straw	Xylosidase from *Weissella cibaria*	10	2.6^e^	70^e^	Enzymatic hydrolysis (37 °C)	[46]
Beechwood xylan	Xylanase from *Mycothermus thermophilus*	12	8.0–8.8^e^	800–880^e^	Enzymatic hydrolysis (65 °C)	[16]
Xylan from corn cobs	Xylanase from *Thermomyces lanuginosus*	8	6.9	345	Enzymatic hydrolysis (45 °C)	[47]
Xylan from data seed	Xylanase from *Aspergillus niger*	4	4.1^e^	411^e^	Enzymatic hydrolysis (38 °C)	[48]
Xylan from vetiver grass	Xylanase from *Aureobasidium melanogenum*	92	4.7	194	Enzymatic hydrolysis (28 °C)	[49]

^a^Reaction time indicates the hydrolysis or fermentation time when XOS concentration reaches the presented value

^b^Xylose is not included

^c^Yields are presented as final concentration (mg/mL) of XOS in fermentation medium

^d^Yields are presented as mass (mg) of XOS obtained from a gram of substrate

^e^These data are measured using liquid chromatogram and others are measured using DNS method

Considerable reducing sugar (1.19 mg/mL at the 24th hour) were produced even without IPTG, which could be attributed to induction of certain saccharides from wheat bran. Further study indicated that XOS concentration induced by wheat bran alone reached 78% of the maximum with 1 mM of IPTG, and adding only a small amount of IPTG (0.02 mM) could lead to the maximal XOS concentration as well as xylanase activity in the fermentation medium (Additional file 1: Fig. S1). Also, *E. coli* BL21(DE3) could still grow and secrete xylanases without additional nitrogen source, suggesting certain components like crude protein of wheat bran could be utilized as nitrogen source (Fig. 6). These results suggested that wheat bran not only acted as substrate for XOS production, but also played an important role in stimulating the secretion of r*Ba*xyl11 and served as nutrient, which is conducive to economical use of extra inducer and to saving cost. Fermentation without wheat bran or using *E. coli* BL21(DE3) containing raw plasmid also produced tiny amounts of reducing sugar, which probably resulted from reducing metabolites secreted by *E. coli* BL21(DE3) (Fig. 3).

Xylanase activity is another factor influencing XOS production. For example, type of nitrogen source actually affected XOS production mainly by changing xylanase activity (Fig. 4), and the increase of activity also contributed to the production in the experiment of optimizing wheat bran concentration (Fig. 5). However, a huge raise in xylanase activity commonly leading to a limited increase in XOS concentration (Fig. 3 and Fig. 6a), suggesting that mere pursuit of high activity or large loading of xylanase could be less effective than expected in large-scale production of XOS. Interestingly, the biomass of *E. coli* BL21(DE3) was very low when the optimal nitrogen source or high wheat bran concentration was employed (Figs. 5a, 6a). It seems that ideal condition for fermentation prejudices bacteria growth but stimulates the accumulation of heterologous proteins.

The GH11 xylanase of *B. agaradhaerens* AC13, BadX, was previously reported to hydrolyze pNPX [31]. However, r*Ba*xyl11 of *B. agaradhaerens* C9 was actually an endo-β-1,4-xylanase, and was not able to act on pNPX according to our results. This difference could be attributed to the change from lysine to glutamic acid of amino acid sequence (Fig. 1b). Xylobiose and xylotriose were the main products by r*Ba*xyl11 acting on wheat bran. Such low-DP XOS commonly present better prebiotic efficacy [32, 33]. Moreover, alkaline environment is optimal for fermentation by *E. coli* containing r*Ba*xyl11, which could prevent hemicellulose from autohydrolysis during heat sterilization thereby avoiding undesired saccharides [34]. Therefore, r*Ba*xyl11 is promising to one-step fermentation for XOS production with advantages in, for example, specificity and stability. The best fermentation temperature is 44.3 °C, which is affect by both of the optimal temperature for catalysis by r*Ba*xyl11 (60 °C) and for growth of *E. coli* (37 °C). Comparing with enzymatic hydrolysis using purified xylanases at high temperature, one-step fermentation employing recombinant *E. coli* adopts mild conditions, which contributes to save energy and cost.

Future research would be made with focus on two components. Firstly, the XOS concentration could be further improved. For example, many other xylanases, substrates, pretreatments or mediums are alternative for fermentation [35]. In particular, the plasmid is worth optimizing. pET22b(+) was used for constructing recombinant *E. coli* in this study. Strictly speaking, it is not an ideal plasmid for secretory expression because only a small part, about 30% according to our measurement, of recombinant proteins was transported into medium. Developing more appropriate plasmids for production is very promising to improve the yield. Secondly, application prospect of such one-step fermentation needs to be evaluated at larger scale. It is obvious that a good XOS yield derived from triangular flasks is no guarantee of the same thing at industrial level. A test using lab-scale fermentation tank is constructive research as well as the first step to promote it from laboratory to factory.

## Conclusions

This work demonstrates that *E. coli* is appropriate for producing XOS with a competitive concentration thereby overcoming the current weakness of one-step fermentation. The critical factor leading to the breakthrough in yield is efficient production of XOS by *E. coli* at high substrate concentration. The optimal conditions, especially pH, for fermentation are highly affected by enzymatic characteristics of the xylanase used. This work provides theoretical basis for overcoming yield and cost challenge, and contributes to the wide adoption of XOS.

## Methods

### Strains, plasmid, and substrates

*B. agaradhaerens* C9 was isolated from saline-alkali soil, and has been maintained in our laboratory since then [36]. *E. coli* DH5α was used for gene cloning and plasmid maintenance. *E. coli* BL21(DE3) was used for gene expression as well as fermentation. pET22b(+), which was previously used for extracellular production of recombinases in many researches [37, 38], was employed for constructing recombinant plasmid.

Arabinoxylan, glucuronoxylan and XOS with DP ranging from 2 to 6 were all purchased from Megazyme (Ireland). Wheat bran was purchased from a flour mill in Huainan city, China. Starch presenting in wheat bran was removed according to the reported method before fermentation [39]. In brief, milled wheat bran was treated with amylase and papain successively. These enzymes were then denatured by boiling for 25 min. After that, wheat bran was washed three times to remove enzymes and starch. The de-starched wheat bran was finally dried and screened through 80 meshes sieve for fermentation and hydrolysis. Xylan content of wheat bran increased from 28.3 to 59.4% after de-starched treatment, which was measured according to the method offered by National Renewable Energy Laboratory [40].

### Heterologous expression and purification

Sequence of *Ba*xyl11 gene is accessible in raw data and it is predicted as xylanase basing on BLAST (https://blast.ncbi.nlm.nih.gov/Blast.cgi). The gene was cloned with forward primer containing *Bam*HI restriction site (5′-CTAGGATCCGCAAATCGTCACCGACAATTCCA-3′) and reverse primer containing *Xho*I restriction site (5′-CCGCTCGAGATTGTTTTTGTCCAAAGTTAT-3′). *Ba*xyl11 gene was ligated into pET22b(+) after digested by endonucleases, and then transferred into *E. coli* DH5α. The validated recombinant plasmid was finally transferred into *E. coli* BL21(DE3) for heterologous expression.

Inducing *Ba*xyl11 gene expression was carried out in LB-ampicillin medium containing 0.6 mM of IPTG at 37 °C, 200 rpm for 6 h. Bacterial cells were then harvested by centrifugation, and were resuspended using Tris–HCl buffer (20 mM, pH8.0) for ultrasonication. After that, soluble cell extract containing r*Ba*xyl11 was collected by centrifuging at 4 °C and was filtered through 0.45-μm filters. r*Ba*xyl11 was purified by affinity chromatography as follows: 5 mL of soluble cell extract was loaded into a Ni–NTA column that was previously equilibrated with binding buffer (20 mM Tri-HCl, 500 mM NaCl, pH 8.0). 12 mL of washing buffer (20 mM imidazole, 20 mM Tri-HCl, 500 mM NaCl, pH 8.0) and 6 mL of elution buffer (250 mM imidazole, 20 mM Tri-HCl, 500 mM NaCl, pH 8.0) were then loaded to remove undesired proteins and to elute r*Ba*xyl11, respectively. Saline ions in eluent were removed by dialysis and r*Ba*xyl11 was finally freeze-dried for reserve.

The purified r*Ba*xyl11 was detected by sodium dodecyl sulfate polyacrylamide gel electrophoresis. In brief, 15 μL of r*Ba*xyl11 solution was mixed with 5 μL of loading buffer and then incubated in boiling water bath for 5 min. After that, the protein solution was load into a polyacrylamide gel (12.5%) for electrophoresis. The gel was dyed using coomassie blue solution to detect the band of protein.

### Enzyme assay

The freeze-dried r*Ba*xyl11 was dissolved using deionized water, and protein concentration was determined according to the absorbancy at 280 nm and the extinction coefficient of r*Ba*xyl11. To measure enzymatic activity, 50 μL of diluted enzyme solution and 100 μL of substrate solution were mixed and incubated at 60 °C, pH 8.0 for 20 min, and the reducing sugars were then measured with dinitrosalicylic acid (DNS) assay and calibration curve using xylose as standard [41]. Kinetic parameters were worked out using Lineweaver–Burk plot according to enzyme activities which were measured with xylan solution whose concentration ranged from 1 to 20 mg/mL [42]. The optimal reaction conditions were investigated by determining enzymatic activities at different temperatures or pH values. To study stability, activities of r*Ba*xyl11 were measured after incubated at 70 °C (Na_2_HPO_4_-NaH_2_PO_4_ buffer, pH 8.0) for different time or incubated in buffers (Na_2_HPO_4_-NaH_2_PO_4_ buffer and Na_2_CO_3_-NaHCO_3_ buffer) with different pH (5.0–10.0) value for one hour.

XOS produced by r*Ba*xyl11 from wheat bran were analyzed using HPIC with a Dionex ICS3000 system. Analytical CarboPac PA10 pellicular anion-exchange resin column (250 by 4 mm) was used for sugar separation. 25 μL of sample was eluted with 250 mM NaOH (1.0 mL/min) at 30 °C and detected by ED 3000 pulsed amperometric detector.

### One-step fermentation

One-step fermentation was carried out as following method if not specifically indicated. Recombinant *E. coli* BL21(DE3) was inoculated into LB-ampicillin medium and grown at 37 °C overnight. Then, appropriate amounts of cells were collected by centrifugation and further diluted to an initial OD_600_ = 1.0 into fermentation medium. After that, ampicillin and IPTG were respectively added to 50 μg/mL and 0.6 mM, and cells were cultured at 37 °C and 200 rpm immediately. After 12 h, supernatant of medium was diluted to measure extracellular xylanase activity and XOS concentration (equivalent xylose) as the method introduced in “One-step fermentation” section. Medium before fermentation was used as control group for measurement of reducing sugar. Each liter of fermentation medium contains: 20 g wheat bran, 4.8 g Na_2_HPO_4_·12H_2_O, 2.65 g KH_2_PO_4_, 4 g (NH_4_)_2_SO_4_, 0.3 g MgSO_4_·7H_2_O, 0.01 g FeSO_4_·7H_2_O, 0.01 g CaCl_2_·2H_2_O and 1 mL trace element solution (0.3 g/L H_3_BO_3_, 0.2 g/L CoCl_2_·6H_2_O, 30 mg/L ZnSO_4_·7H_2_O, 30 mg/L MnCl_2_·4H_2_O, 30 mg/L NaMoO_4_·2H_2_O, 20 mg/L NiCl_2_·6H_2_O and 10 mg/L CuSO_4_·5H_2_O). The pH value of fermentation medium was adjusted to 7.0 before sterilization. To investigate the optimal nitrogen source, NH_4_SO_4_ in initial medium was replaced with equal-mass tryptone, casein acids hydrolysate, yeast extract, urea, beef extract, NH_4_NO_3_, NaNO_3_ or glycine for fermentation and the medium without additional nitrogen source was used as control. All experiments were performed in triplicate.

### Measurement of biomass

Growth of *E. coli* was measured by dilute plate method. 100 μL of medium after fermentation was sampled and diluted 10^5^–10^7^ times using NaCl solution (0.9%). 200 μL of diluted medium was spread onto a LB-ampicillin agar plate and cultured at 37 °C overnight. Colony forming unit (CFU) was finally counted to evaluate the biomass of *E. coli* in medium after fermentation.

### Optimization of XOS production

Response surface methodology was employed to optimize XOS production by one-step fermentation using Box-Behnken experimental design [20]. Nitrogen source concentration, pH and fermentation temperature were selected for optimization. Experimental design was provided in Table 2, and the range of these factors was chosen basing on pretest.

### Bioinformatic and statistical analysis

Signal peptide was predicted using Signalp 4.0 server (http://www.cbs.dtu.dk/services/SignalP-4.0/). Glycoside hydrolase family was predicted using dbCAN meta server (http://bcb.unl.edu/dbCAN2/blast.php). Sequence alignment was carried out using DNAMAN v6 software package. Statistical analysis was carried out using t-test (least significant difference).

## Supplementary Information

**Additional file 1:** Fig. S1  Effect of IPTG concentration on XOS yield and xylanase activity of fermentation.

## Data Availability

Raw data are available at https://doi.org/10.6084/m9.figshare.16779289.v1

## Abbreviations

CFU: Colony forming unit
DNS: 3,5-Dinitrosalicylic acid
DP: Degree of polymerization
GH: Glycoside hydrolase
HPIC: High pressure ion chromatography
IPTG: Isopropyl-1-thio-β-D-galactopyranoside
pNPX: 4-Nitrophenyl-beta-D-xylopyranoside
XOS: Xylo-oligosaccharides
