# Supplementary figures and images for: One-step fermentation for producing xylo-oligosaccharides from wheat bran by recombinant Escherichia coli containing an alkaline xylanase
Source: BMC Biotechnol. 2022 Feb 5;22:6. doi: 10.1186/s12896-022-00736-8 (PMC8817556; doi:10.1186/s12896-022-00736-8)

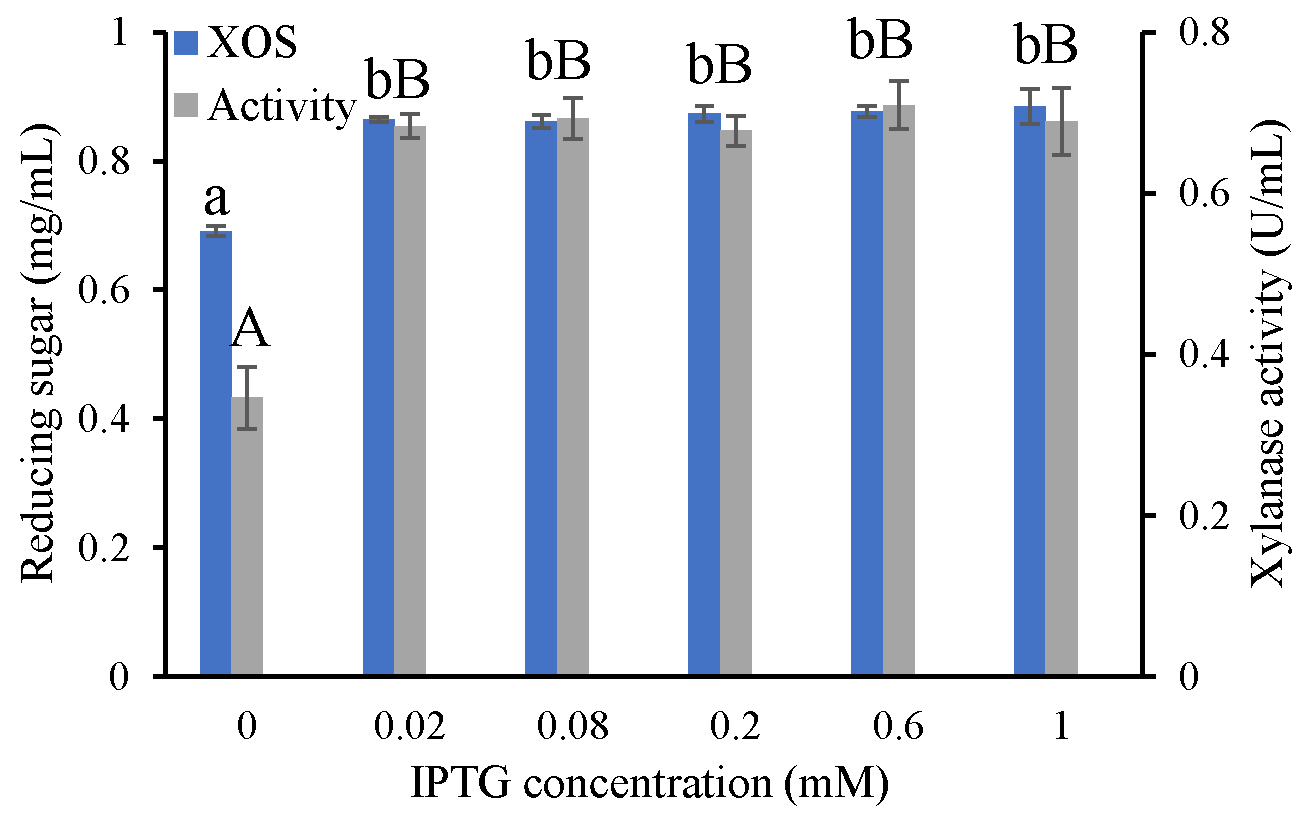

Supplement: Supplementary file 1 — Additional file 1: Fig. S1 Effect of IPTG concentration on XOS yield and xylanase activity of fermentation. [file 12896_2022_736_MOESM1_ESM.tif]
